# Characteristics of pediatric central venous catheter malfunction and subsequent catheter-related deep vein thrombosis

**DOI:** 10.1016/j.rpth.2026.107011

**Published:** 2026-10-12

**Authors:** Natasha S. Bala, Zaineb Boulil, Emily Krava, Char Witmer, Sarah H. O’Brien, Lingyun Ji, Guy Young, Julie Jaffray

**Affiliations:** 1 Center for Cancer and Blood Disorders, Department of Pediatrics, Phoenix Children’s Hospital, Phoenix, Arizona, USA; 2 Research Administration Department, Rady Children’s Hospital, San Diego, California, USA; 3 Department of Pediatrics, University of California San Diego, La Jolla, California, USA; 4 Division of Hematology, Children’s Hospital of Philadelphia, Philadelphia, Pennsylvania, USA; 5 Division of Pediatric Hematology/Oncology/Bone Marrow Transplant, Nationwide Children's Hospital, Ohio State University College of Medicine, Columbus, Ohio, USA; 6 Center for Child Health Equity and Outcomes Research, Abigail Wexner Research Institute, Nationwide Children's Hospital, Columbus, Ohio, USA; 7 Department of Population and Public Health Sciences, Keck School of Medicine, University of Southern California, Los Angeles, California, USA; 8 Hemostasis and Thrombosis Center, Cancer and Blood Disorders Institute, Children's Hospital Los Angeles, University of Southern California Keck School of Medicine, Los Angeles, California, USA; 9 Division of Pediatric Hematology/Oncology, Department of Pediatrics, University of California San Diego, La Jolla, California, USA; 10 Peckham Center for Cancer and Blood Disorders, Rady Children’s Hospital, San Diego, California, USA

**Keywords:** central venous catheters, child, complications, risk factors, venous thrombosis

## Abstract

**Background:**

Central venous catheters (CVCs) are increasingly placed in children; however, malfunction and deep vein thrombosis (DVT) are reasons for frequent removal.

**Objectives:**

The objective of this study was to evaluate variables associated with CVC malfunction and, subsequently, the development of CVC-related DVT (CR-DVT).

**Methods:**

This subanalysis of the multicenter, prospective Clot Incidence Rates in Central Lines study enrolled children aged 6 months to <18 years with newly placed peripherally inserted central catheters or tunneled lines from 2013 to 2018. Time from CVC placement to first malfunction was analyzed in the full cohort using univariable and multivariable Weibull regression. Among CVCs that malfunctioned, time from first malfunction to CR-DVT was analyzed to assess tissue plasminogen activator (t-PA) use and subsequent thrombosis.

**Results:**

Malfunction occurred in 380/1951 CVCs (19.5%). In multivariable analysis, prior CVC use (hazard ratio [HR], 1.51; 95% CI, 1.19-1.90; *P* = .001), multilumen catheters (HR, 2.89; 95% CI, 2.30-3.65; *P* < .001), and >2 insertion attempts (HR, 2.21; 95% CI, 1.42-3.45; *P* < .001) were associated with a higher risk of malfunction, whereas catheter size >4 French and interventional radiology placement were associated with a lower risk. Among malfunctioning CVCs, 22/380 (5.8%) developed CR-DVT, with a median time to CR-DVT of 6 days after malfunction. Increasing t-PA use showed a nonsignificant trend toward higher subsequent CR-DVT.

**Conclusion:**

CVC malfunction is common and associated with several modifiable catheter-related factors. Six percent of malfunctioning CVCs develop CR-DVT, and repeated t-PA use and persistent CVC dysfunction may warrant further evaluation for underlying thrombosis.

## Essentials

• Central venous catheters (CVCs) are frequently placed in children but have a risk of malfunctioning.
• This multicenter study sought to identify risk factors associated with CVC malfunction.
• Prior CVCs, smaller-lumen and multilumen catheters, and multiple insertion attempts were associated with a higher risk of malfunction.
• These potentially modifiable factors can be mitigated to prevent premature CVC removal.
• CVC malfunction variables related to CVC type, insertion or patient characteristics can be mitigated to minimize risk of malfunction.
• Frequent use of t-PA for CVC malfunction could forewarn CVC-related DVT.

## 1 Introduction

Central venous catheters (CVCs) are essential tools in pediatric care, used for a range of diagnostic and therapeutic purposes, from short-term intravenous treatments to long-term outpatient administration of chemotherapy, total parenteral nutrition, or apheresis [1]. Caution is advised for CVC insertion, and placement should not be based on “convenience of care” or “provider habit” [2]. Despite their utility, CVCs carry a substantial risk of complications, including infection, occlusion, breakage, and thrombosis, which can lead to early catheter removal in more than one-third of pediatric patients [1], [3], [4]. Replacing a CVC poses additional morbidity, including further anesthesia exposure, infection risk, and vascular damage, as well as additional healthcare expenditure [5], [6], [7].

Among these complications, catheter malfunction, defined as the inability to aspirate or infuse through a CVC due to partial or complete occlusion, is often underrecognized [8], [9]. Once considered a mechanical issue solely, catheter malfunction is now emerging as a potential contributor to the pathogenesis of catheter-related deep vein thrombosis (CR-DVT). The pathophysiological link is thought to involve impaired fluid flow through a blocked catheter and prolonged dwell time of irritant infusates, such as chemotherapy, which can promote endothelial injury and thrombus formation [10]. CVC malfunction has consistently been associated with CR-DVT. Earlier studies demonstrated a 65% increase in the odds of CR-DVT with malfunctioning catheters, and this association was further supported by the prospective Clot Incidence Rates in Central Lines (CIRCLE) study, which reported a nearly 6-fold higher risk of CR-DVT with malfunctioning CVCs [10], [11]. Malfunctioning CVCs can subsequently lead to catheter removal if they do not respond to thrombolytic agents or more invasive procedures, such as fibrin sheath stripping or balloon angioplasty [12], [13].

Despite these concerning consequences, risk factors for CVC malfunction and its role as a precursor to CR-DVT remain poorly characterized in pediatric populations. Variations in catheter type (eg, peripherally inserted central catheters [PICCs] vs tunneled lines [TLs]), insertion technique, underlying medical diagnoses, and infusion protocols likely contribute to malfunction risk but have not been rigorously examined in a large, prospective pediatric cohort. In this subanalysis of the CIRCLE study [11], we aimed to identify patient- and CVC-related risk factors associated with CVC malfunction. We also examined the risk of subsequent CR-DVT among CVCs with reported malfunction. By clarifying these relationships, our findings aim to inform more targeted CVC care practices and preventive strategies to reduce thrombosis risk in pediatric patients.

## 2 Methods

### 2.1 Study design

This study is a subanalysis of the CIRCLE study [11], a prospective, multicenter, observational cohort study whose primary objective was to evaluate the incidence and risk factors for CR-DVT in children with newly placed PICCs or TLs. The study was conducted from October 2013 to June 2018 at 4 major pediatric hospitals in the United States [11]. The original CIRCLE study [11] was approved by the institutional review board at each participating site, with a waiver of informed consent granted.

### 2.2 Study population

Participants in the original CIRCLE study [11] were children aged 6 months to <18 years with a newly placed PICC or TL. Participants were randomly selected for enrollment at the time of CVC insertion using daily or weekly reports of newly placed CVCs at each institution; differences in demographics, medical history, or reason for CVC placement among subject populations were not considered. An interim analysis showed that more subjects with PICCs were enrolled because of the high volume of PICC placements. Thus, the remaining TLs were enrolled consecutively, and newly placed PICCs were randomly selected to achieve an approximately 1:1 ratio of TLs to PICCs. The full study methodology has been described previously [11]. Infants under 6 months of age were excluded due to distinct developmental differences in hemostasis that could confound thrombotic outcomes. Similarly, children with nontunneled CVCs, such as temporary internal jugular, subclavian, or femoral lines, were excluded, as the primary aim of the CIRCLE study [11] was to assess complications associated with longer-term catheter use. The decision on the type of catheter placed (PICC or TL) was made by the participant’s clinical care team based on institutional protocols and clinical indications.

For this subanalysis, the full cohort of the CIRCLE study [11] was screened for inclusion to evaluate CVC-related risk factors associated with CVC malfunction. Participants were monitored prospectively through review of the electronic medical record (EMR) for the development of CVC malfunction, which was defined *a priori*. Malfunction was defined as either (1) a CVC blockage requiring administration of tissue plasminogen activator (t-PA) or (2) a CVC with a mechanical malfunction, including those with a fibrin sheath or medication precipitation. Malfunctions were identified by reviewing EMR data for medication precipitation in nursing or provider notes and radiographic data for the presence of a fibrin sheath. CVCs reported to be damaged (eg, a crack or tear) or malpositioned were not classified as malfunctions for the purpose of this subanalysis. To evaluate the association between CVC malfunction and CR-DVT, only CVCs with malfunctions were included. Malfunctioning CVCs reported after a CR-DVT diagnosis were excluded.

### 2.3 Study procedures

Participants were enrolled in the CIRCLE study [11] based on reports of patients with newly placed PICCs or TLs at each participating institution. Enrolled participants were followed prospectively until CVC removal or the end of the 5-year study period, whichever occurred first. Individuals with multiple CVCs placed during the study period were captured as separate enrollments, provided they had not been diagnosed with a CR-DVT. Data from participants with multiple CVC enrollments were linked using a unique participant identification number assigned at study entry. CVC malfunction was assessed through prospective, serial review of EMR documentation, including bedside nursing and vascular access/CVC nursing documentation, physician notes, and pharmacy records for t-PA administration, and recorded separately for each CVC enrollment and analyzed at the catheter level. The diagnosis of CR-DVT was confirmed by radiographic imaging performed at the discretion of the clinical care team based on symptoms or concerns for deep vein thrombosis (DVT).

Data were collected by trained study personnel through review of the EMR using standardized case report forms in Research Electronic Data Capture. Details of the collected data elements have been published previously [11]. For this analysis, participant-related data included sociodemographic variables, medical history, current use of total parenteral nutrition or anticoagulation, and the presence of catheter-associated bloodstream infection (CLABSI). CVC-related data included CVC characteristics (type, size, number of lumens, material, and length) and placement procedure details (vein accessed, tip location, insertion attempts, placement under ultrasound [US] guidance, and placement using interventional radiology [IR] techniques).

### 2.4 Statistical analysis

Descriptive statistics were calculated for sociodemographic, clinical, and CVC characteristics of all participants. Mean ± SD or median (IQR) and frequency (percentage) were used to summarize continuous and categorical variables, respectively, as appropriate. For the primary analysis, we conducted survival analysis, modeling time from CVC placement to the first malfunction as the outcome. CVCs were censored at catheter removal, the end of study follow-up, or the last known observation without malfunction. We fit univariable and multivariable Weibull regression models, a parametric survival modeling approach that allows modeling of time-varying hazard functions. Variables with *P* ≤ .20 in univariable analyses were considered for inclusion in the multivariable model and retained using backward elimination, keeping variables with *P* ≤ .10. From these models, HRs and corresponding 95% CIs were obtained. All *P* values were 2-sided, and those <.05 were considered statistically significant. We also generated a cumulative incidence curve stratified by catheter type (PICC vs TL) to compare malfunction incidence between line types over time. These estimates reflect malfunction risk among devices that remained in place. For participants who experienced multiple malfunctions, only the first event was included in all survival analyses, including the Weibull regression models and the cumulative incidence curve.

In the secondary analysis, the time from the first malfunction to radiographically confirmed CR-DVT was assessed among CVCs with at least 1 malfunction. Participants who had CR-DVT without a prior CVC malfunction event during the same enrollment period were excluded from this analysis. CVCs were censored at catheter removal, the end of follow-up, or 6 months after malfunction. Given the limited number of CR-DVT events, this analysis was considered exploratory. Associations between the number of t-PA doses and subsequent CR-DVT were assessed using univariable Weibull regression.

## 3 Results

### 3.1 Participant characteristics

Of the 2006 CVC placements enrolled, 1996 were available for analysis, consistent with the original CIRCLE study [11]. Of these, 1951 CVCs from 1734 unique participants were analyzed in this study. Reasons for exclusion included previous enrollment with a subsequent diagnosis of CR-DVT (*n* = 26), inability to confirm CVC placement dates and/or malfunction (*n* = 16), current treatment for CR-DVT (*n* = 2), and inability to verify eligibility (*n* = 1). The median age of participants at the time of CVC insertion was 6.5 years (IQR, 2.7-12.4). Most CVC placements were in participants of White race (*n* = 950, 48.7%), and more than one-third were of Hispanic ethnicity (*n* = 653). Additionally, the most common underlying medical diagnosis was cancer (*n* = 793, 40.6%). Sixty-four percent of CVCs were PICCs (*n* = 1249), and 36% were TLs (*n* = 702). The most common TL type was a port-a-cath (*n* = 479, 68%). Full demographic, clinical, and CVC-related characteristics are summarized in Tables 1 and 2.

**Table 1:** Comparison of baseline sociodemographic and clinical characteristics by central venous catheter malfunction.

Sociodemographic/clinical characteristics	At least 1 malfunction (*n* = 380) *n* (%)	No malfunctions (*n* = 1571) *n* (%)
**Age at CVC insertion (y), median (IQR)**	5.07 (2.3-12.0)	6.77 (2.9-12.5)
**Age group (y)**		
0.5-1	31 (8.2)	109 (6.9)
>1-5	159 (41.8)	544 (34.6)
>5-10	69 (18.2)	372 (23.7)
>10-18	121 (31.8)	546 (34.8)
**Sex**		
Female	179 (47.1)	731 (46.5)
Male	201 (52.9)	840 (53.5)
**Ethnicity**		
Hispanic/Latino	148 (38.9)	505 (32.1)
Non-Hispanic	119 (31.3)	683 (43.5)
Not listed	113 (29.7)	383 (24.4)
**Race**		
White	175 (46.1)	775 (49.3)
Asian	26 (6.8)	98 (6.2)
Black or African American	33 (8.7)	193 (12.3)
Native Hawaiian or Pacific Islander	1 (0.3)	6 (0.4)
American Indian or Native Alaskan	1 (0.3)	14 (0.9)
Unknown/Other	144 (37.9)	485 (30.9)
**Past medical history**		
Cancer	198 (52.1)	595 (37.9)
Congenital heart disease	32 (8.4)	95 (6.0)
Current infection (other than CLABSI)	80 (21.1)	430 (27.4)
Cystic fibrosis	5 (1.3)	85 (5.4)
History of a venous thrombus	23 (6.1)	108 (6.9)
Inflammatory bowel disease	6 (1.6)	32 (2.0)
Juvenile rheumatoid arthritis	1 (0.3)	4 (0.3)
Lupus	0 (0)	5 (0.3)
Metabolic or mitochondrial disease	25 (6.6)	67 (4.3)
Nephrotic syndrome	2 (0.5)	13 (0.8)
History of prior CVC placement	156 (41.1)	498 (31.7)
Surgery in last 30 d	90 (23.7)	335 (21.3)
No medical history	28 (7.4)	248 (15.8)
**CLABSI**	28 (7.4)	121 (7.7)
**Use of TPN**	80 (21.1)	329 (20.9)
**Use of anticoagulation**		
Yes	22 (5.8)	74 (4.7)
No	248 (65.3)	939 (59.8)
Unknown/missing	110 (28.9)	558 (35.5)

CLABSI, central line-associated bloodstream infection; CVC, central venous catheter; TPN, total parenteral nutrition.

**Table 2:** Comparison of baseline central venous catheter-related characteristics by central venous catheter malfunction.

CVC characteristics	At least 1 malfunction (*n* = 380) *n* (%)	No malfunctions (*n* = 1571) *n* (%)
**Type of CVC**		
PICC	231 (60.8)	1018 (64.8)
TL	149 (39.2)	553 (35.2)
**CVC size**		
≤4 French	209 (55.0)	932 (59.3)
>4 French	169 (44.5)	626 (39.8)
Unknown	2 (0.5)	13 (0.8)
**No. of lumens**		
1 (single lumen)	201 (52.9)	1032 (65.7)
>1 (multilumen)	171 (45.0)	452 (28.8)
Unknown/other	8 (2.1)	87 (5.5)
**CVC material**		
Polyurethane	223 (58.7)	660 (42.0)
Silicone	65 (17.1)	255 (16.2)
Unknown/other	92 (24.2)	656 (41.8)
**CVC length**		
Mean ± SD	25.4 ± 9.22	25.8 ± 10.4
Missing	173 (45.5)	631 (40.2)
**Vein accessed**		
Lower extremity vein	14 (3.7)	59 (3.8)
Neck vein	88 (23.2)	380 (24.2)
Upper extremity vein	270 (71.1)	1103 (70.2)
Unknown/other	8 (2.1)	29 (1.8)
**CVC tip location**		
SVC	129 (33.9)	431 (27.4)
Prior to SVC	17 (4.5)	37 (2.4)
SVC/right atrium junction	172 (45.3)	917 (58.4)
IVC	5 (1.3)	28 (1.8)
Right atrium	51 (13.4)	115 (7.3)
Unknown/other	6 (1.6)	43 (2.7)
**Insertion attempts**		
1 or 2	246 (64.7)	1152 (73.3)
>2	20 (5.3)	46 (2.9)
Unknown	114 (30.0)	373 (23.7)
**US guidance**		
No	104 (27.4)	303 (19.3)
Yes	245 (64.5)	1159 (73.8)
Unknown	31 (8.2)	109 (6.9)
**IR guidance**		
No	285 (75.0)	889 (56.6)
Yes	94 (24.7)	676 (43.0)
Unknown	1 (0.3)	6 (0.4)

CVC, central venous catheter; IR, interventional radiology; IVC, inferior vena cava; PICC, peripherally inserted central catheter; SVC, superior vena cava; TL, tunneled lumen; US, ultrasound.

### 3.2 CVC malfunction

Overall, 19.5% (*n* = 380) of CVCs had at least 1 malfunction during the study period. The median time from CVC placement to the first malfunction was 20 days (IQR, 7.8-57). The median insertion time for CVCs with malfunctions was 97 days (IQR, 27.5-205.5), compared with 35 days (IQR, 12-194) for those without malfunctions. Most CVCs had 1 malfunction (*n* = 219, 57.6%), followed by 2 to 3 malfunctions in 118 CVCs (31%), and ≥4 malfunctions in 43 (11.3%) CVCs. PICCs were twice as likely to malfunction as TLs (HR, 2.14; 95% CI, 1.71-2.68; *P* < .001). The estimated 12-month cumulative incidence of malfunction was 55.2% (SE, 5.4%; 95% CI, 43.3%-64.5%). The incidence of malfunction was 60.6% (SE, 7.9%; 95% CI, 41.5%-73.5%) for PICCs and 51.5% (SE, 7.7%; 95% CI, 33.8%-64.4%) for TLs within 12 months of CVC placement (*P* < .001; Figure).

**Figure:**
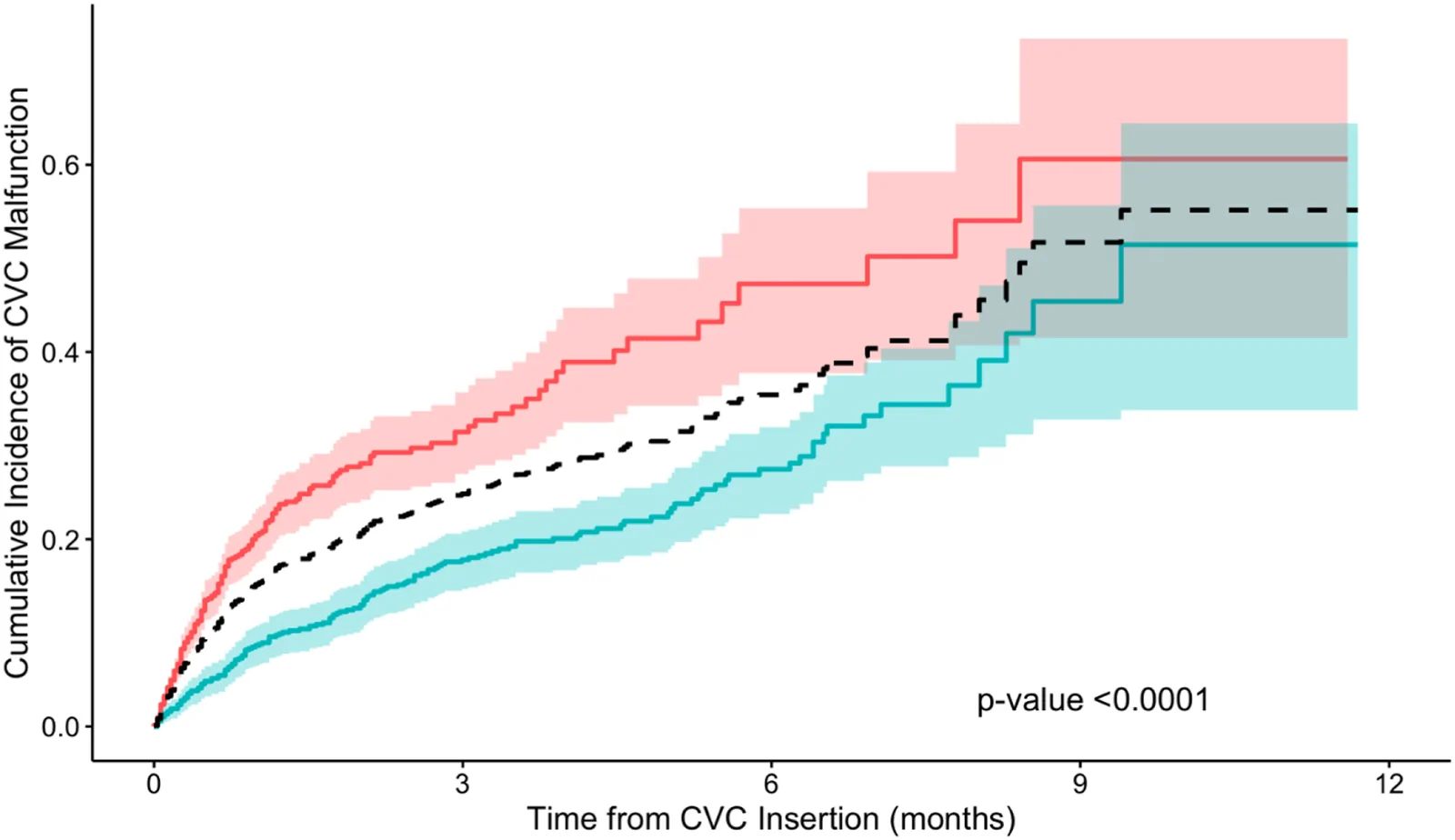
Comparison of central venous catheter (CVC) malfunction events between peripherally inserted central catheters and tunneled lines within 12 months after insertion.

Details of univariate and multivariate models for demographic, clinical, and CVC-related risk factors and malfunction are summarized in Supplementary Tables S1 and S2. In the multivariable analysis, prior CVC use (HR, 1.51; 95% CI, 1.19-1.90; *P* = .001), multilumen catheters (HR, 2.89; 95% CI, 2.30-3.65; *P* < .001), and >2 insertion attempts (HR, 2.21; 95% CI, 1.42-3.45; *P* < .001) were independently associated with a significantly increased risk of CVC malfunction. Catheter size >4 French (F; HR, 0.31; 95% CI, 0.24-0.39; *P* < .001) and IR placement technique (HR, 0.37; 95% CI, 0.28-0.49; *P* < .001) were independently associated with a significantly lower risk of CVC malfunction. CLABSI was also associated with a lower risk of CVC malfunction (HR, 0.60; 95% CI, 0.41-0.88; *P* < .001; Supplementary Table S3).

### 3.3 CLABSI and malfunction

Among malfunctioning CVCs, CLABSI was identified in 18.7% (*n* = 71), and nearly one-third were removed due to CLABSI (*n* = 24, 33.8%). The median dwell time for CVCs with and without CLABSI was 134 days (IQR, 64-223) and 83.5 days (IQR, 24-205), respectively. Among CVCs without malfunction, CLABSI occurred in 7.8% (*n* = 122), and nearly half were removed due to CLABSI (*n* = 62, 50.8%). The median dwell time for CVCs with and without CLABSI was 72 days (IQR, 34-175) and 30 days (IQR, 11-195), respectively.

### 3.4 CVC-related DVT

CR-DVT was diagnosed in 22 of 380 (5.8%) CVCs with malfunctions. The median time from the first CVC malfunction to CR-DVT was 6 days (IQR, 1-18.5). The HR for developing CR-DVT was higher among CVCs requiring more t-PA doses (*P* = .15; Table 3). Among patients with a DVT, the median number of t-PA doses was 2 (range, 0 to ≥4), compared with 1 (range, 0 to ≥4) among those with CVC malfunction but no DVT.

**Table 3:** Univariable analysis of tissue plasminogen activator doses and association with catheter-related deep vein thrombosis.

**Total doses of t-PA**	CVC-associated DVT (*n* = 22) *n* (%)	HR (95% CI)	*P* value
0	2 (9.1)	1.0	
1	6 (27.3)	0.87 (0.16, 4.57)	.87
2	4 (18.2)	1.23 (0.21, 7.23)	.81
3	2 (9.1)	1.15 (0.15, 8.68)	.89
>4	8 (36.4)	3.37 (0.66, 17.27)	.11

CVC, central venous catheter; DVT, deep vein thrombosis; HR, hazard ratio; t-PA, tissue plasminogen activator.

## 4 Discussion

In this subanalysis of the prospective, multicenter CIRCLE study [11], one-fifth of nearly 2000 newly placed CVCs malfunctioned, underscoring the clinical relevance of this complication. Our findings showed that prior CVC use, multilumen CVCs, and multiple attempts at CVC insertion were associated with increased malfunction, whereas CVCs placed under IR guidance and those using a catheter size greater than 4F were associated with decreased malfunction. An unexpected finding was that CLABSIs were associated with lower rates of CVC malfunction. The results also showed that 6% of malfunctioning CVCs developed a CR-DVT, and CVCs requiring repeated doses of t-PA demonstrated a trend toward CR-DVT, though this did not reach statistical significance.

Our observed CVC malfunction rate of 19.5%, with PICCs having twice the malfunction rate of TLs, is similar to prior pooled estimates reported by Ullman et al. [14]. The study reported malfunction incidence rates of 2.2 and 0.85 per 1000 catheter days for PICCs and TLs, respectively [14]. Although that study [14] quantified malfunction by catheter days, both studies suggest that malfunction is common across cohorts and that the risk is markedly higher in patients with PICCs than in those with TLs. In addition, 2 single-center studies reported higher CVC malfunction rates with PICCs than with TLs in patients with hematologic and oncologic disorders [15], [16]. The higher malfunction rate in PICCs likely reflects anatomical and technical factors, such as PICCs traversing a longer intravascular pathway through smaller vessels that are more susceptible to obstruction. Understanding these differences is particularly important since PICCs are typically placed more often than TLs in hospitalized children [7], [17].

In our study, multilumen CVCs were associated with an increased risk of malfunction, consistent with findings in other studies that warrant CVC removal due to occlusion [3], [18], [19], [20]. This may be because individual lumens within multilumen catheters have smaller internal diameters than those of a comparable single-lumen catheter, increasing susceptibility to occlusion and malfunction. CVCs with larger catheter sizes have a higher risk of thrombosis [21], likely due to the increased caliber occupying a larger area of the vessel lumen and obstructing blood flow; however, no studies have confirmed a relationship between CVC size and the risk of malfunction. In our study, CVCs with smaller lumens (≤4F) were associated with higher malfunction rates. These findings are consistent with other studies reporting higher rates of occlusion in smaller-lumen PICCs (15.2% for 3F vs 3.7% for 4F vs none for 5F) [22], and a similar trend was observed in a pilot study of children on hemodialysis, where smaller CVCs had shorter dysfunction-free survival rates, though not statistically significant [23]. Higher malfunction rates in smaller-lumen CVCs may result from occlusion of the narrow lumen by medication precipitation or increased shear stress and sluggish fluid flow. Inappropriate flushing or locking protocols may also contribute to CVC lumen occlusion in smaller-caliber catheters [24].

Additionally, our study found that CVCs placed using the IR technique were less likely to lead to malfunction. This contrasts with previous studies that found no difference in CVC occlusion or malfunction rates between IR and surgical techniques [25], [26]. These studies were limited to specific line types; for example, Chau et al. [25] included only femoral vein tunneled CVCs, and Martin et al. [26] included only ports. Our study included PICCs, TLs, and ports and showed reduced CVC malfunction rates with IR techniques, even after adjusting for US use. This warrants further exploration of the benefits of an IR-guided approach to pediatric catheter placement beyond image guidance alone, such as shorter placement times or higher rates of technical success [27], likely related to less manipulation of the vasculature.

CLABSI was associated with a lower risk of malfunction. This likely reflects the impact of clinical management rather than a truly protective effect. Catheters with CLABSI are typically removed after an infection is identified, thereby reducing the risk of CVC malfunction. Alternatively, frequent access to CLABSI-infected lines and instilling alcohol locks for treatment may aid in maintaining their patency.

This prospective study of a diverse group of pediatric patients found that repeated need for t-PA may be a marker of persistent catheter dysfunction, which could reflect either an underlying process that predisposes to thrombosis or an early manifestation of an evolving CR-DVT. This statistically nonsignificant finding may reflect the small number of postmalfunction CR-DVTs (*n* = 22) captured in this subanalysis and warrants further exploration in future studies. However, frequent t-PA use should prompt further investigation for thrombosis if not already pursued. In a retrospective multicenter cohort study of critically ill children with PICCs and nontunneled CVCs using the Pediatric Health Information System administrative dataset, t-PA use was significantly associated with hospital-acquired venous thromboembolism [28]. Similarly, a recent meta-analysis of hospitalized pediatric patients found an increased risk of CR-DVT with CVC malfunction (odds ratio, 1.65; 95% CI, 1.07-2.54), although CVC malfunction was not explicitly defined [10]. CVC occlusion can result from a fibrin sheath, intraluminal clot, mural thrombus, or CVC-associated DVT [12]. For managing CVC malfunction after ruling out obstruction from mechanical causes or drug precipitation, the use of thrombolytic agents such as t-PA is considered the standard of care [12]. The American College of Chest Physicians guidelines recommend using t-PA as a local thrombolytic agent for up to 2 doses in children and neonates with blocked CVCs, and, if there is no response, imaging is recommended to rule out CVC-associated DVT [29]. Hence, recurrent t-PA use may be a harbinger of underlying CVC-associated DVT, and it is important to distinguish the 2, since management differs between intermittent thrombolytic therapy for CVC malfunction and antithrombotic agents for CVC-associated DVT. Despite the association between CVC malfunction and thrombosis, prophylactic anticoagulation for occluded CVCs is not considered standard of care.

Our study had some limitations. The sampling strategy was modified during the study to increase the representation of TLs relative to PICCs; although eligible PICCs were randomly selected and TLs were enrolled consecutively, this change may have affected the cohort’s representativeness and introduced selection bias. Data on the use of heparin, normal saline flushes, or other agents for flushing CVCs, which may affect CVC malfunction, were not captured prospectively, even though all participating institutes routinely used heparin locks or flushes. There were 671/1249 (53.7%) PICCs and 99/702 (14.1%) TLs placed using IR guidance. Although IR-guided placement was associated with a lower risk of malfunction in our analysis, we cannot determine whether this reflects differences in catheter type, patient characteristics (such as body mass index), procedural issues, or other unmeasured confounders. Malfunctions due to fibrin sheath formation or medication precipitation could not be differentiated since not all malfunctioning lines were imaged in this study. However, t-PA use was considered a surrogate marker for CVC malfunction. The timing between multiple t-PA doses varied considerably across participants, ranging from hours to weeks, and the study was not designed to systematically capture or analyze the interval between sequential t-PA doses or its impact on malfunction. US screening for DVT was not performed routinely at set intervals but rather at the discretion of the provider. Hence, whether participants who received multiple doses of t-PA had already developed a DVT prior to its recurrent use could not be ascertained. Rates of subsequent DVT after CVC malfunction were low, and therefore further analysis of risk factors associated with DVT could not be investigated. Moreover, this study took place at 4 large tertiary pediatric hospitals within a single country, where practices related to CVC care, resource availability, and patient population may differ, thus limiting the generalizability of the results.

In conclusion, our study demonstrates high rates of CVC malfunction and identifies several potentially modifiable patient- and CVC-related risk factors. Prior to CVC insertion in children, CVC type and insertion technique should be considered to help inform strategies to potentially reduce malfunction. The burden of central line malfunction includes treatment interruptions and the need for thrombolytic agents. Incorporating standardized CVC placement and best care practices, including monitoring for dysfunction, can help reduce premature CVC removal and replacement. Future studies should explore whether prevention strategies aimed at reducing CVC malfunction can also lower the incidence of CR-DVT in children.
