# Supplementary material for: Characteristics of pediatric central venous catheter malfunction and subsequent catheter-related deep vein thrombosis
Source: Res Pract Thromb Haemost. 2026 Oct 12;10(7):107011. doi: 10.1016/j.rpth.2026.107011 (PMC13760109; doi:10.1016/j.rpth.2026.107011)
Supplement: Supplementary file 1 [file mmc1.docx]

**Supplementary Table S1. Univariable Models**

|  | **Hazard Ratio (95% C.I.)** | **p-value** |
| --- | --- | --- |
| **Cancer** |  |  |
| No | 1.0 |  |
| Yes | 0.804 (0.64, 1.004) | 0.052 |
| **Cancer Type** |  |  |
| No cancer | 1.0 |  |
| Leukemia/Lymphoma | 0.86 (0.67, 1.09) | 0.22 |
| Other cancer | 0.74 (0.55, 0.99) | 0.04 |
| **Congenital heart disease** |  |  |
| No | 1.0 |  |
| Yes | 2.18 (1.45, 3.27) | 0.0002 |
| **Current infection (other than CLABSI)** |  |  |
| No | 1.0 |  |
| Yes | 1.09 (0.85, 1.41) | 0.48 |
| **Cystic Fibrosis** |  |  |
| No | 1.0 |  |
| Yes | 0.48 (0.19, 1.17) | 0.11 |
| **History of a venous thrombus** |  |  |
| No | 1.0 |  |
| Yes | 0.85 (0.52, 1.39) | 0.53 |
| **Inflammatory bowel disease** |  |  |
| No | 1.0 |  |
| Yes | 0.98 (0.41, 2.32) | 0.96 |
| **Juvenile Rheumatoid Arthritis** |  |  |
| No | 1.0 |  |
| Yes | 1.62 (0.35, 7.59) | 0.54 |
| **Metabolic or Mitochondrial Disease** |  |  |
| No | 1.0 |  |
| Yes | 1.93 (1.24, 3.0) | 0.003 |
| **Nephrotic Syndrome** |  |  |
| No | 1.0 |  |
| Yes | 0.97 (0.24, 3.89) | 0.96 |
| **History of prior CVC placement** |  |  |
| No | 1.0 |  |
| Yes | 1.33 (1.07, 1.66) | 0.0099 |
| **Surgery last 30 days** |  |  |
| No | 1.0 |  |
| Yes | 1.13 (0.89, 1.45) | 0.31 |
| **No Medical History** |  |  |
| No | 1.0 |  |
| Yes | 0.67 (0.46, 0.99) | 0.044 |
| **CLABSI** |  |  |
| No | 1.0 |  |
| Yes | 0.72 (0.50, 1.04) | 0.083 |
| **Use of TPN** |  |  |
| No | 1.0 |  |
| Yes | 1.52 (1.17, 1.97) | 0.0015 |
| **Use of Anticoagulation** |  |  |
| No  Yes | 1.0  1.31 (0.78, 2.18) | 0.31 |
| **Type of CVC**  PICC    TL  **CVC Size** | 1.0  0.47 (0.37, 0.58) | <0.0001 |
| ≤4 | 1.0 |  |
| > 4 | 0.5 (0.40, 0.63) | <0.0001 |
| Unknown | 0.36 (0.10, 1.22) | 0.1 |
| **Number of Lumens** |  |  |
| 1 | 1.0 |  |
| > 1 | 2.28 (1.83, 2.83) | <0.0001 |
| Unknown | 0.38 (0.19, 0.78) | 0.008 |
| **CVC Material** |  |  |
| Polyurethane | 1.0 |  |
| Silicone | 0.78 (0.59, 1.04) | 0.096 |
| Unknown | 0.32 (0.25, 0.42) | <0.0001 |
| **CVC Length** |  |  |
| Median [Min, Max] | 0.99 (0.98, 1.01) | 0.69 |
| **Vein Accessed**  Lower extremity vein  Neck vein  Upper extremity vein  Unknown  **CVC Tip Location** | 1.0  0.39 (0.22, 0.73)  0.72 (0.41, 1.28)  0.78 (0.32, 1.89) | 0.0027  0.266  0.581 |
| SVC | 1.0 |  |
| Prior to SVC | 1.64 (0.91, 3.00) | 0.103 |
| SVC/right atrium junction | 0.75 (0.59, 0.95) | 0.015 |
| IVC | 1.02 (0.39, 2.60) | 0.969 |
| Right atrium | 1.29 (0.92, 1.82) | 0.135 |
| Unknown | 1.28 (0.39, 4.10) | 0.680 |
| **Insertion Attempts**  1 or 2  >2  Unknown  **Ultrasound Guidance** | 1.0  1.99 (1.28, 3.10)  1.43 (1.13, 1.81) | 0.0023  0.0024 |
| No | 1.0 |  |
| Yes | 1.12 (0.89, 1.42) | 0.36 |
| Unknown | 0.98 (0.65, 1.48) | 0.92 |
| **IR Guidance** |  |  |
| No | 1.0 |  |
| Yes | 0.75 (0.59, 0.97) | 0.03 |
| Unknown | 0.36 (0.05, 2.84) | 0.33 |
|  |  |  |
|  |  |  |
|  |  |  |
|  |  |  |
|  |  |  |
|  |  |  |
|  |  |  |

**Supplementary Table S2. Multivariable Model (including covariates identified from the univariable modeling as having p-values ≤ 0.2)**

|  | **Hazard Ratio (95% C.I.)** | **p-value** |
| --- | --- | --- |
| **Cancer Type** |  |  |
| No cancer | 1.0 |  |
| Leukemia/Lymphoma | 1.02 (0.75, 1.39) | 0.879 |
| Other cancer | 1.15 (0.82, 1.63) | 0.421 |
| **Congenital heart disease** |  |  |
| No | 1.0 |  |
| Yes | 0.97 (0.59, 1.59) | 0.918 |
| **Cystic Fibrosis** |  |  |
| No | 1.0 |  |
| Yes | 0.59 (0.24, 1.46) | 0.256 |
| **Metabolic or Mitochondrial Disease** |  |  |
| No | 1.0 |  |
| Yes | 1.20 (0.74, 1.96) | 0.463 |
| **History of prior CVC placement** |  |  |
| No | 1.0 |  |
| Yes | 1.39 (1.09, 1.78) | 0.009 |
| **No Medical History** |  |  |
| No | 1.0 |  |
| Yes | 0.69 (0.45, 1.08) | 0.107 |
| **CLABSI** |  |  |
| No | 1.0 |  |
| Yes | 0.54 (0.37, 0.79) | 0.002 |
| **Use of TPN** |  |  |
| No | 1.0 |  |
| Yes | 1.19 (0.89, 1.58) | 0.230 |
| **Vein Accessed** |  |  |
| Lower extremity vein | 1.0 |  |
| Neck vein | 0.92 (0.41, 2.04) | 0.829 |
| Upper extremity vein | 0.91 (0.44, 1.87) | 0.495 |
| Unknown | 0.67 (0.22, 2.09) | 0.792 |
| **CVC Size** |  |  |
| ≤ 4 | 1.0 |  |
| > 4 | 0.54 (0.34, 0.85) | 0.008 |
| Unknown | 0.56 (0.16, 1.90) | 0.354 |
| **Number of Lumens** |  |  |
| 1 | 1.0 |  |
| > 1 | 3.15 (2.46, 4.04) | <0.0001 |
| Unknown | 0.86 (0.40, 1.83) | 0.696 |
| **CVC Material** |  |  |
| Polyurethane | 1.0 |  |
| Silicone | 1.07 (0.75, 1.53) | 0.712 |
| Unknown | 0.42 (0.31, 0.58) | <0.0001 |
| **Insertion Attempts** |  |  |
| 1 or 2 | 1.0 |  |
| > 2 | 2.12 (1.36, 3.30) | 0.001 |
| Unknown | 1.77 (1.35, 2.32) | <0.0001 |
| **IR Guidance** |  |  |
| No | 1.0 |  |
| Yes | 0.47 (0.34, 0.65) | <0.0001 |
| Unknown | 0.17 (0.02, 1.81) | 0.141 |
| **CVC tip Location** |  |  |
| SVC | 1.0 |  |
| Prior to CVC | 1.19 (0.56, 2.54) | 0.657 |
| SVC/right atrium junction | 0.91 (0.71, 1.18) | 0.495 |
| IVC | 0.55 (0.17, 1.77) | 0.317 |
| Right atrium | 1.33 (0.92, 1.93) | 0.124 |
| Unknown | 0.85 (0.35, 2.07) | 0.713 |

**Supplementary Table S3. Final Multivariable Model (covariates identified by performing backward variable selection on previous multivariable model to include those with p-values ≤ 0.1)**

|  | **Hazard Ratio (95% C.I.)** | **p-value** |
| --- | --- | --- |
| **History of prior CVC placement** |  |  |
| No | 1.0 |  |
| Yes | 1.51 (1.19, 1.90) | 0.001 |
| **CLABSI** |  |  |
| No | 1.0 |  |
| Yes | 0.60 (0.41, 0.88) | 0.009 |
| **CVC Size** |  |  |
| ≤ 4 | 1.0 |  |
| > 4 | 0.31 (0.24, 0.39) | <0.0001 |
| Unknown | 0.22 (0.08, 0.64) | 0.006 |
| **Number of Lumens** |  |  |
| 1 | 1.0 |  |
| > 1 | 2.89 (2.30, 3.65) | <0.0001 |
| Unknown | 0.58 (0.28, 1.22) | 0.156 |
| **Insertion Attempts** |  |  |
| 1 or 2 | 1.0 |  |
| > 2 | 2.21 (1.42, 3.45) | 0.0005 |
| Unknown | 1.79 (1.39, 2.32) | <0.0001 |
| **IR Guidance** |  |  |
| No | 1.0 |  |
| Yes | 0.37 (0.28, 0.49) | <0.0001 |
| Unknown | 0.16 (0.02, 1.53) | 0.111 |
